# Performance Optimization Approach of Polymer-Modified Asphalt Mixtures with PET and PE Waste

**DOI:** 10.3390/polym16233308

**Published:** 2024-11-27

**Authors:** Assel Jexembayeva, Marat Konkanov, Lyazat Aruova, Akpan Kirgizbayev, Lailya Zhaksylykova

**Affiliations:** 1 Department of Industrial and Civil Construction Technology, Eurasian National University, Astana 010000, Kazakhstan; 2 ENU Lab, Eurasian National University, Astana 010000, Kazakhstan; konkanov_md@enu.kz

**Keywords:** modified asphalt, PE waste, PET, rheological property, physical properties, asphalt mixtures

## Abstract

Road infrastructure sustainability and pavement performance may be increased by using waste materials like polyethylene terephthalate (PET) and polyethylene waste (PE waste) in polymer-modified asphalt mixtures. As seen by a more pronounced rise in the softening point, which exceeds 110 °C with 8% PE waste, PET was found to improve the tensile strength, resistance to cracking, and thermal stability of asphalt mixes. PE waste also increases ductility up to 4% PE waste, beyond which the combination becomes more brittle, and dramatically decreases penetration, strengthening the asphalt’s resistance to deformation. Additionally, bitumen treated with PE waste is more workable than asphalt without PET, even though bitumen treated with PE waste had a viscosity of up to 4500 Pa. Complex shear modules decreased as the PE waste and PET content increased. PET, on the other hand, increases the binder’s overall stiffness, elasticity, and tensile strength. Nevertheless, when PET content rises, ductility steadily decreases. Previous studies concentrated on the effects of each component separately, and this paper fills this knowledge gap by investigating the combined effects of PET and PE waste. The results indicate that the highest compressive strength (7.5 MPa) was obtained with 6% PE + 2% PET, while the highest tensile strength (1.40 MPa) was achieved with a balanced mix of 4% PE waste + 4% PET. Additionally, the viscosity of asphalt is increased by PET and PE waste, enhancing its performance at high temperatures. These findings demonstrate how combining PET and PE waste improves the mechanical and thermal characteristics of asphalt, providing a balance between stiffness and flexibility, a crucial feature for durable road materials under a variety of circumstances.

## 1. Introduction

For thousands of years, asphalt has been utilized for waterproofing and sophisticated road construction. For infrastructure development, its versatility, robustness, and recyclability are very important. The goal of contemporary asphalt technology is to increase sustainability and environmental performance [1].

Numerous issues, each with distinct characteristics and causes, such as heat, fatigue, and edge cracking, have an impact on the performance of asphalt. The stress caused by vehicles can seriously erode pavement integrity, leading to rutting. Aggregate particle loss, or raveling, is brought on by age or abrasion, whereas surface quality is compromised by potholes, which are frequently caused by moisture and freeze–thaw cycles [2,3]. However, due to the extreme overloading and rapid growth in traffic volume, there are serious problems with asphalt pavements. Early deterioration is a common result of asphalt pavement on many expressways being unable to support traffic demands right after construction [4]. Addressing these challenges through monitoring and intervention measures is critical to ensuring the structural integrity and functionality of asphalt pavements during their service life [5].

The use of polymer waste is an innovative way to improve asphalt and address plastic pollution. Although a large amount of plastic continues to end up in landfills or is burned, polymer waste enhances asphalt performance and provides a sustainable solution for plastic waste [6]. The rheological characteristics of asphalt binders, which have a major influence on rutting and other issues, are closely linked to these challenges [7]. Engineers and scientists employ modifiers, such as fillers, fibers, polymers, and other additives, to improve binder qualities and solve or minimize these difficulties [8].

While polymer-modified asphalt has many benefits, there are also certain disadvantages to consider. The higher cost of polymers can be problematic for projects with limited funding. In addition, inadequate performance, diminished longevity, and premature pavement erosion can result from polymer and asphalt incompatibilities [9].

Engineers can improve quality control standards by gaining a deeper understanding of how modifiers affect the performance of asphalt binders. This study explores the use of PET and PE waste in polymer-modified asphalt mixes to enhance performance and support sustainable infrastructure. Aging resistance, fracture resistance, and crack resistance are important areas of attention. Despite the benefits of employing PET and PE waste, prior research has primarily concentrated on the individual effects of each modifier, resulting in a dearth of information regarding their combined effects within Kazakhstan’s specific traffic and climatic conditions.

### Literature Review

Asphalt is a strong and resilient material that can survive heavy traffic and varying weather conditions, making it essential for road infrastructure. Nevertheless, seasonal variations and growing traffic cause pavement degradation on a regular basis [10]. In response to this challenge, researchers have concentrated on improving asphalt by using polymers. This approach is a promising way to improve the quality and longevity of road surfaces. Polymer-enriched asphalt presents many advantages that greatly improve pavement sustainability and performance [3,11].

The numerous benefits of polymer-modified asphalt (PMA) include its increased resilience to rutting, cracking, and fatigue in high-traffic and adverse weather conditions. The structural integrity of pavements is preserved by the increased flexibility and durability provided by polymers, which reduces the need for regular maintenance and repairs [12]. Furthermore, the use of polymers in asphalt encourages eco-friendly activities. Recycled polymer waste not only promotes sustainable resource management and waste reduction but also enhances pavement performance, supporting the circular economy [13]. PMA was created in response to the difficulties presented by growing traffic volumes, aiming to enhance asphalt’s performance, longevity, and adherence to aggregates. These challenges place excessive pressure on conventional pavements, leading to their shorter lifespans and more frequent repairs. To improve performance and durability, PMA manufacturers mix asphalt with polymers. Typically, the percentage of polymer by weight is 3–7% [14,15,16,17].

Although environmental and socioeconomic factors make repairing roads challenging, considerable efforts have been made to reduce the occurrence of pavement failures. In order to improve bitumen’s strength, stability, and longevity, researchers have explored methods of modifying it. These projects have successfully utilized a variety of waste materials, such as crumb rubber, nylon, polymer waste [18], polyethylene [19], polypropylene [20], PET [21], and polystyrene [22]. The goal of these modifications is to enhance pavement sustainability and performance.

This study attempts to improve important bituminous properties that are essential for building roads. With advantages including improved elastic recovery, a higher softening point, enhanced viscosity, cohesive strength, and ductility, bitumen polymer matrices are a commonly employed modification technique [23]. Yan et al. [10] propose using an anti-aging agent derived from amorphous poly alpha olefin (APAO) to further optimize the compressive performance of polymer-modified asphalt. Additionally, studies suggest that rubberized asphalt with 20% rubber particles holds the potential for creating crumb rubber asphalt, which can resolve issues related to workability and storage stability.

According to Xin et al. [24], adding PE (polyethylene) composites to asphalt binders considerably raises the composite shear modulus and enhances mechanical performance. Desidery et al. [25], for example, report that the addition of synthetic waxes to recycled polyethylene (RPE) improves the composite shear modulus and rutting resistance factor of modified asphalt, leading to superior rheological properties at both high and low temperatures. Yao et al. [26] found that adding rubber and PE to asphalt enhances its performance and resistance to rutting in cold weather. PE is a linear polymer that is created when ethylene monomers undergo free radical polymerization [27].

By stiffening the mixture and increasing its resistance to deformation, polyethylene [28] (PE) is added to asphalt to improve its performance at low temperatures and to prevent rutting. This improves durability under traffic loads by changing the rheological properties of the asphalt binder and raising its complex modulus. Polyethylene with a high or medium density can be employed for this purpose [29].

The increased global usage of polyethylene terephthalate (PET) has made PET waste an increasingly important environmental concern. PET is mechanically recycled into secondary materials, which is a crucial strategy to mitigate its carbon impact. This approach conserves resources and eliminates the need for the production of virgin PET, thus involving less energy and having less environmental effect. However, it often results in inferior materials that are limited in their use [30].

According to the study conducted by Faizan Mushtaq et al. [11], polymer-modified asphalt mixes that use PET and PE waste are a promising means of improving the sustainability of road materials. This finding justifies the wider use of these environmentally friendly construction approaches, as they satisfy global sustainability goals and guarantee the preservation of performance and safety requirements. Therefore, it can be concluded that using recycled materials in construction can support sustainable development objectives and foster research into innovative ways to use waste resources in engineering [11]. 

While prior research has explored polymer modifiers such as PET and PE waste in asphalt mixtures, it has mostly focused on the short-term performance of individual polymers under moderate circumstances. The combined effects of PET and PE on the long-term mechanical, thermal, and rheological characteristics of asphalt remain understudied, especially in the context of arid regions. Unmodified asphalt is more likely to develop problems like cracking in the winter and rutting in the summer. By adding elasticity and viscosity to the binder, polymer additives can greatly improve its characteristics and the pavement’s overall performance, making it more resilient. 

The purpose of the current study is to examine how different PET and PE waste concentrations affect the mechanical, thermal, and rheological properties of asphalt mixes treated with polymers. This research highlights critical gaps in existing studies on asphalt modification with polymer additives, focusing on the combined use of PET and PE waste. Below are the key points:

1. Combined polymer effects: Most studies examine either PET or PE waste. Investigations into their combined impact may reveal synergistic benefits that enhance asphalt’s mechanical and thermal properties;
2. Limited research on performance in harsh environments: Previous research mainly assesses asphalt mixes treated with polymers in mild environments. The present study endeavors to investigate the effectiveness of modified asphalt under adverse weather conditions and with high traffic volumes, therefore answering the demand for materials that are more resilient in such environments;
3. Environmental sustainability: This research aims to reduce plastic waste and extend the lifespan of asphalt pavements, which may reduce the need for routine maintenance and lessen the overall environmental effect.

## 2. Materials and Methods

### 2.1. Asphalt Material

The type of asphalt used in this study was Asphalt 70 Penetration Grade (Atirau, Kazakhstan). This type of asphalt is extensively employed in road building; its physical properties are presented in Table 1. The combination of hardness and flexibility renders Asphalt 70 Penetration Grade suitable for a wide range of climatic conditions and traffic loads. Asphalt 70 Penetration Grade has a penetration value of 70–100 at 25 °C, indicating its hardness or softness. Higher penetration numbers indicate softer asphalt. This type of asphalt has a lower viscosity than tougher grades of asphalt, allowing for simpler mixing and application at low temperatures. It also has good thermal susceptibility, which means it can adapt to temperature fluctuations without significantly altering its qualities. 

### 2.2. PET and PE Waste

The polyethylene terephthalate (PET) utilized in this study as the first modifier was sourced from KMI Company in Almaty, Kazakhstan. Due to its favorable properties, polyethylene waste is ideal for various applications, including asphalt modification. The second modifier, PE waste, is typically obtained from discarded plastic items, including packaging, containers, and plastic bags. PE waste must be carefully chosen to guarantee purity and quality. The PE waste used in this investigation came from Atyrau, Kazakhstan. The properties of PE waste and PFT are presented in Table 2.

Cleaning is necessary to remove any pollutants, dirt, or other impurities from the PE waste. To remove particles from the plastic waste, this step usually entails washing it with water and detergents. Next, it is dried to make sure there is no moisture left, as moisture might impede the asphalt mixing process.

To increase the PE waste’s compatibility with the asphalt binder, it is cleaned and then shredded or ground into tiny pieces. The shredded particles must be small enough to allow for uniform dispersion in the asphalt mixture yet not so finely divided as to degrade during the mixing process. Typical particle sizes range from 1 to 5 mm in diameter.

### 2.3. Production of PET and PE Waste-Modified Asphalt

This study used a wet procedure to create asphalt modified with PET and PE waste. Before 400 g of asphalt and polymer waste were mixed in a high-speed shear mixer running at 5000 rpm (AE500S-H high-shear machine (Angni, Zhengzhou, China)), the asphalt was first heated to 140 °C in an oven. To guarantee uniform blending, the polymer modifier concentrations were adjusted to 0%, 2%, 4%, 6%, and 8% by weight of the asphalt. The procedure was conducted at a constant temperature of 185 °C. The modifier was completely dissolved and uniformly distributed throughout the asphalt mixture after an hour of high-speed shearing. After mixing, a stabilizer was added, and to ensure total homogeneity, the mixture was sheared one more time. Once the mixing was completed, the asphalt–polymer blend was allowed to cool gradually to room temperature, ensuring proper curing and stabilization of the material before further testing. To guarantee that the polymer was distributed uniformly throughout the asphalt mixture, high-speed shearing was performed in the laboratory at 5000 rpm. 

Standard asphalt plants are equipped with mixing devices that heat and mix asphalt at high temperatures, similar to the 185 °C employed in the laboratory, for large-scale road construction projects. Numerous plants have the ability to mix at high shear or may be equipped with mixers that operate at high torque, simulating the shearing effects observed in laboratory settings that are smaller in size. Adjustments in the design and operation of these industrial mixers can help ensure that the right balance of shear forces and temperature control is maintained during mixing.

Large-scale road construction projects may not be able to benefit from smaller-scale high-speed shear mixing due to the volume of materials required; however, industrial asphalt factories frequently use these high-torque mixers to guarantee that asphalt and polymers are blended uniformly, achieving the same level of homogeneity and performance as observed in the laboratory. Large-scale infrastructure projects and the commercial manufacturing of polymer-modified asphalt can both benefit from the scaling up of this process. 

Following conventional procedures outlined in [31], the asphalt–polymer blends were prepared using a wet technique. The composites were then crushed using a mechanical compactor to obtain consistent density and structural integrity. The asphalt–polymer mixture was heated to around 140 °C and poured into cylindrical molds with a diameter of 100 mm and a height of 63.5 mm. The samples were compressed to a hydraulic pressure of around 600 kPa in order to simulate normal field compaction conditions.

The compactor worked at 30 blows per minute, giving each sample 75 blows on each side or 50 gyrations when the Superpave technique was employed. Depending on the method used, the complete compaction process takes two to three minutes for each sample, making sure the mixture reaches the necessary void content and density. The mixture temperature was kept between 135 °C and 140 °C during compaction to provide the best possible bonding between the asphalt and polymer modifiers and avoid premature cooling. Furthermore, the process of compaction was conducted in a controlled setting, where the relative humidity was kept within the range of 50% and 60% in order to reduce the potential impact of moisture on the mechanical characteristics of the asphalt mixture. Before undergoing additional mechanical testing, the molds were allowed to cool for one to two hours at ambient temperature to provide adequate curing and stability following compaction. This compaction process ensured that the modified asphalt would possess the mechanical qualities required for dependable operation in a variety of high-traffic and environmental scenarios.

**Figure 1:**
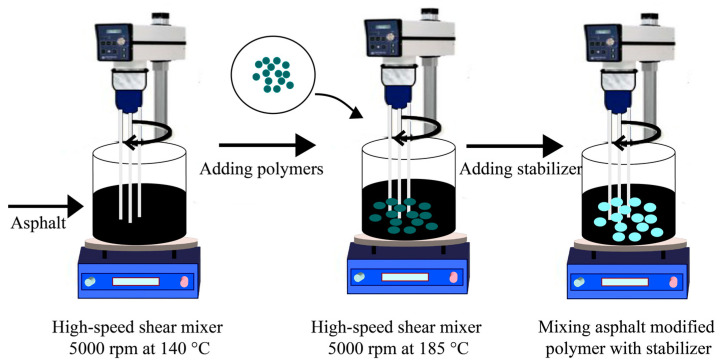
The preparation of asphalt-modified polymer.

To approximate real-world application circumstances, great attention was paid to regulating the compaction process’s temperature, pressure, and number of blows. At a temperature over 140 °C, compaction was carried out to preserve workability and prevent excessive air spaces in the samples. Prior to conducting any mechanical tests, the specimens were subjected to room temperature cooling following compaction to guarantee adequate stability of the asphalt–polymer composites. Figure 1 illustrates the preparation process of asphalt-modified polymer. 

### 2.4. Experimental Methods

Fourier Transform Infrared Spectroscopy (FTIR) was used to examine the chemical composition of materials and study how a substance absorbs infrared light. This device consists of an infrared source, beam splitter, interferometer, sample holder, and detector. FTIR may reveal data on the chemical structure of polymer-modified asphalt by recognizing its functional groups and chemical bonds based on their distinct infrared absorption patterns. The device provides high-resolution spectra (1 and 0.4 cm^−1^) by scanning the material between 4000 and 500 cm^−1^. This degree of detail enables the accurate identification of molecular alterations and interactions in the asphalt.

X-ray diffraction (XRD) was utilized to examine the materials used in this investigation, which included asphalt, PET, and PE waste. To ensure uniformity, the samples were coarsely powdered before being placed on the XRD sample holder. A D8 ADVANCE universal diffractometer (Bruker, Ettlingen, Germany) with a copper anode (wavelength: 1.5406 Å) was used for the XRD studies. Diffraction patterns were obtained in a 2θ range between 10° and 70°, with the operational parameters set at 40 kV and 40 mA. The crystalline structure and phase composition of the polymer-modified asphalt could be thoroughly examined through a precise analysis made possible by the XRD scanning speed of 1°/min and step size of 0.02.

To prepare the samples for the analysis, the following procedures were employed:

- PET and PE Waste: To create a pellet, the materials were finely powdered and combined with potassium bromide (KBr) in a 1:100 ratio. This technique reduced scattering effects during analysis and enabled the best possible presentation of the material;
- Asphalt: The asphalt samples were either combined with KBr to form pellets or melted and molded into thin films on appropriate substrates.

The softening point test is necessary to ascertain the temperature at which a material softens under particular circumstances. When evaluating the thermal stability and rheological characteristics of asphalt binders treated with polymers, this test is especially important. The softening point, according to [32], offers information about how a material reacts to high temperatures, enabling an assessment of the material’s performance under various environmental conditions.

In order to evaluate the flow and deformation behavior of asphalt binders, it is essential to use rheological testing. The penetration test [33] gauges the binder’s hardness by determining how deeply, under controlled circumstances, a standard needle can penetrate it. The material’s viscosity is evaluated using the viscometer test* [34], which provides an indication of how resistant it is to flow at high temperatures. An important measure of a binder’s flexibility and tensile strength is how far it can stretch before breaking, which is assessed by the ductility test [35].

Another crucial instrument for analyzing the viscosity and elastic characteristics of asphalt binders, especially at medium to high temperatures, is the dynamic shear rheometer (DSR). This tool aids in the investigation of the rheological properties of binders that have been altered using materials like plastic waste. The test is performed over a temperature range of 50 °C to 75 °C to measure the temperature sensitivity of these modified binders. The DSR is a crucial test for forecasting long-term durability because it provides detailed insights into the binder’s performance under various temperature conditions and traffic loads by examining these qualities.

To test compressive strength, the resultant mixture was placed into cylindrical molds; for tests of tensile strength, it was poured into dog-bone-shaped molds. The specimens were 150 mm long, 20 mm wide at the narrowest point, and 40 mm thick. Cylindrical specimens had dimensions of 100 mm in height and diameter. Prior to testing, the specimens underwent a 24 h curing period at room temperature to guarantee adequate bonding and durability. Compressive strength was measured with a Universal Testing Machine (UTM) fitted with a compression platen in accordance with [36]. The cylindrical samples were subjected to a continuous axial load by the UTM until failure occurred, at which point the maximum load was noted. By dividing the greatest force by the specimen’s cross-sectional area, the compressive strength was computed.

In testing facilities, short curing times (24 h) are frequently employed to preserve uniformity across samples and guarantee that the circumstances are reproducible. This makes it easier to compare findings with those of other research using comparable procedures. Thus, the materials can settle and establish bonds that are indicative of their early-stage performance over the course of 24 h at ambient temperature. This is important for routine laboratory tests that measure the mechanical characteristics of the material immediately.

Tensile strength tests were conducted using [37]. The dog-bone-shaped specimens were subjected to an ongoing tensile strain by the same UTM, which was equipped with tensile grips, until they broke. The highest load was then divided by the cross-sectional area at the specimen’s narrowest point to obtain the tensile strength.

To guarantee consistency, every test was carried out at room temperature (25 °C), and three repetitions of each test were required to obtain accurate and reliable results. For analysis, the average compressive and tensile strength values were noted. This approach made it possible to thoroughly analyze the mechanical performance of asphalt mixes that were altered with different ratios of PE waste and PET, both alone and together, under specific conditions.

## 3. Results

### 3.1. Modification Mechanism of PET and PE Waste-Modified Asphalt 

#### 3.1.1. Chemical Structure Analysis of PET, PE Waste, and Asphalt

**Figure 2:**
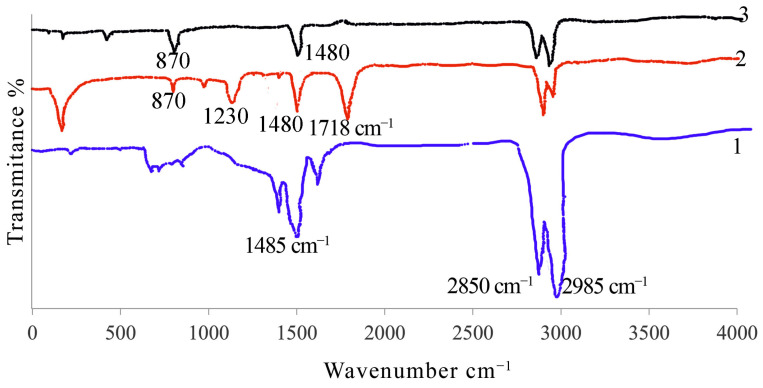
The FTIR of PET, PE waste, and the asphalt they modified: unmodified asphalt (1), PET (2), and PE waste (3).

Several important findings regarding the chemical composition and interactions within the material were obtained from the FTIR study of modified asphalt containing PET and PE waste. The findings in Figure 2 demonstrate that identical distinctive bands, which denote shared chemical structures in these materials, are displayed by the modified asphalt as well as the PET and PE waste. The FTIR spectrum of unmodified asphalt exhibits distinctive peaks that indicate the basic chemical linkages found in the material. The notable peaks include the methyl C-H bond stretching vibrations at 2985 cm^−1^ and 2850 cm^−1^, which are representative of the hydrocarbon structure in asphalt and are linked to naphthenes and alkanes. Furthermore, two prominent absorption peaks that are intrinsic to the unaltered asphalt, at 1485 cm^−1^ and 1376 cm^−1^, are evident in the saturated C-H bond bending vibration.

New characteristics are introduced when comparing the PET spectrum to untreated asphalt. The prominent peak at 1230 cm^−1^, which corresponds to the symmetric C–C–H stretching in the aromatic ring of PET, indicates the presence of aromatic carbon structures. Furthermore, distinctive molecular structures that contribute to a particular chemical profile in the PET-modified asphalt are shown by its different peaks at 870 and 1480 cm^−1^, respectively. These peaks, especially the one at 1230 cm^−1^, draw attention to the aromatic components of PET, which probably affect the properties of the asphalt by adding stiffness and rigidity. Despite this, the methyl C-H stretching vibrations (in the range of 2985 to 2850 cm^−1^) are evident, indicating that PET does not completely change the fundamental hydrocarbon structure of asphalt.

PE waste does not considerably change the core hydrocarbon structure, as shown by the FTIR spectra of PE waste, which exhibits the same methyl C-H bond stretching peaks at 2985 cm^−1^ and 2850 cm^−1^ as those seen in unmodified asphalt. However, the PE waste-modified asphalt also demonstrates a characteristic absorption peak at 1480 cm^−1^, indicating that the binder’s saturated C-H bond bending vibration persists. Since PE waste lacks a distinctive peak at 1252 cm^−1^, it does not provide any noteworthy aromatic compounds compared to PET. PE waste shares absorption peaks with unmodified asphalt at 870 and 1480 cm^−1^, suggesting even more structural similarities.

The FTIR analysis concludes that whereas PET and PE waste provide asphalt with distinct chemical properties, they both mostly maintain the core hydrocarbon structure of the binder. While PE waste maintains a chemical profile more similar to that of unaltered asphalt, PET adds aromatic rings that affect the chemical characteristics of the asphalt. With PET boosting rigidity and PE waste enhancing flexibility, these variations have different effects on the mechanical and thermal characteristics of the asphalt binders.

#### 3.1.2. X-Ray Diffraction (XRD)

**Figure 3:**
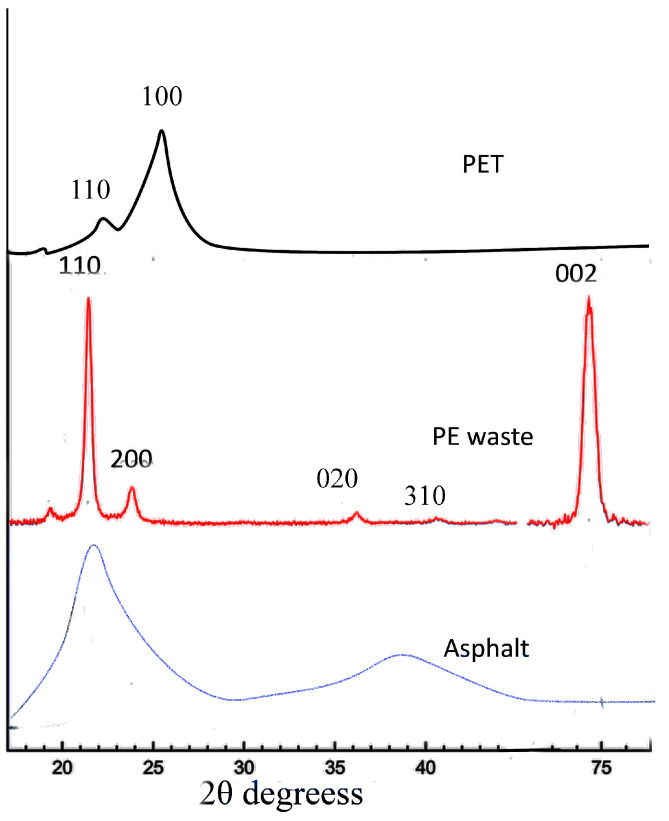
X-ray diffraction of PE waste, PET, and asphalt.

In order to provide comprehensive details regarding X-ray diffraction (XRD) patterns for PE waste, PET, and Asphalt 70 Penetration Grade (Figure 3), comprehensive experimental procedures and results from XRD analysis must be included. It should be noted that XRD analysis uses measurements of the diffraction patterns of X-rays passing through a sample to determine the crystalline structure and phase compositions of materials.

PE waste usually possesses both crystalline and amorphous areas, indicating a semi-crystalline structure. PE often exhibits significant diffraction peaks in its XRD patterns, which correlate to its crystalline regions. Particularly, PE waste exhibits prominent diffraction peaks at around 21.5° and 23.9° (2θ), which correspond to orthorhombic polyethylene’s (110) and (200) crystal planes. Furthermore, the XRD spectrum’s wide hump, which is usually seen between 15° and 25° (2θ), indicates that the material contains amorphous material. In a similar vein, the semi-crystalline PET material shows different diffraction peaks linked to its crystalline areas. The diffraction pattern of PET has peaks at approximately 17°, 22.5°, and 25.5° (2θ), corresponding to the crystal planes (010), (110), and (100), respectively.

By comparison, Asphalt 70 Pen has low crystallinity and is mostly amorphous. The XRD pattern, which shows a mostly amorphous structure, is characterized by wide humps rather than sharp peaks. In the Asphalt 70 Pen XRD spectrum, the wide hump at 22° (2θ) clearly illustrates the material’s amorphous nature. This amorphous quality, although sacrificing some structural stiffness in comparison to more crystalline materials, adds to the material’s flexibility and resistance to different climatic conditions. 

The XRD analysis highlights the interaction between the crystalline and amorphous areas of these materials, which greatly affects their mechanical and physical characteristics. The data on the distinction between crystalline and amorphous content aid in forecasting the conduct of these substances in various scenarios, offering significant perspectives for their utilization in sectors like construction and research.

### 3.2. Effect of PET and PE Waste on Physical Properties of Asphalt

**Figure 4:**
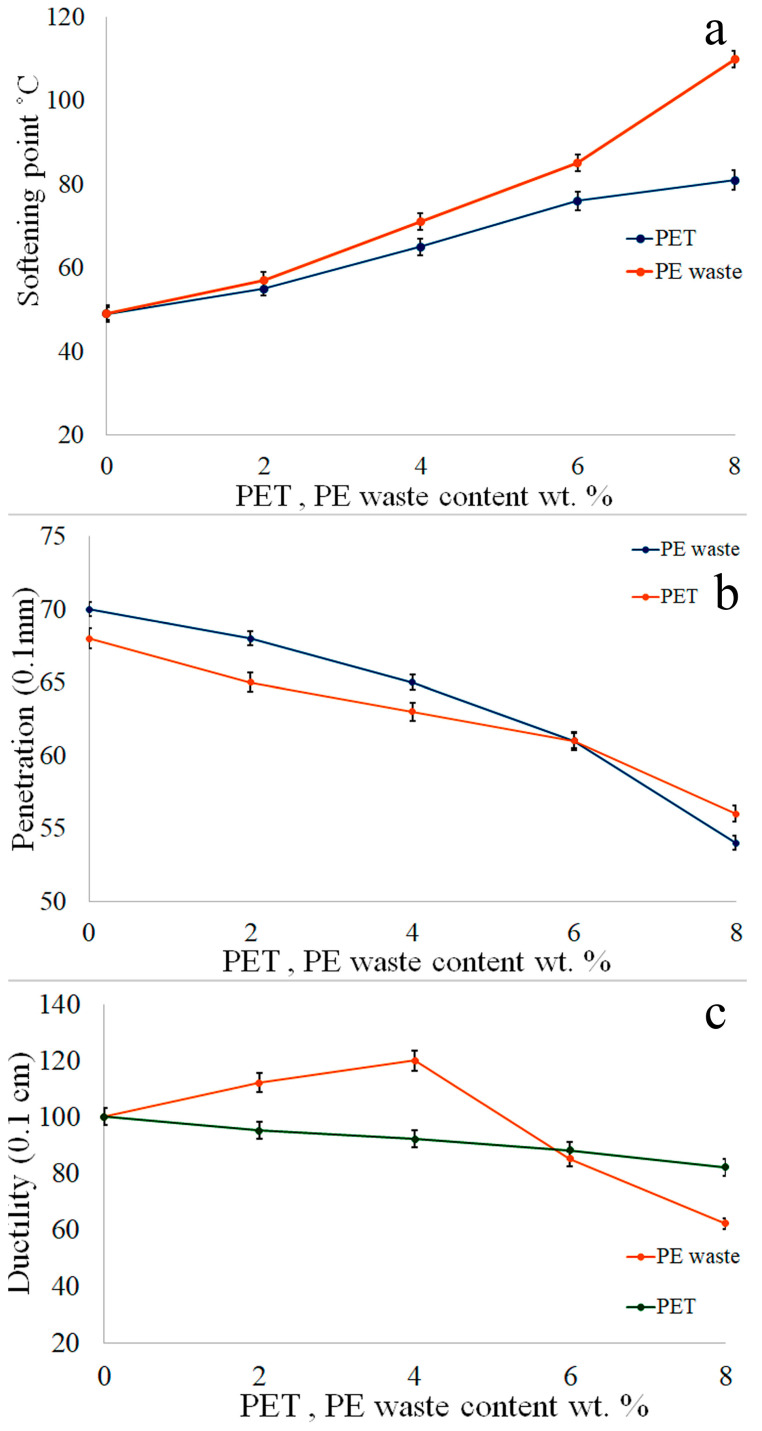
Test results regarding PE-modified asphalt’s physical properties (softening point (**a**); penetration (**b**); ductility (**c**)).

The softening points of an asphalt binder are commonly used to evaluate the polymer-modified asphalt’s storage stability. Figure 4a shows the test results for the physical parameters of PET and PE-waste-treated asphalt. With the increasing proportion of PET and PE waste, the softening point also increases. In the case of PE waste, the pace of increase is more noticeable. Although the softening point of PET-modified asphalt increases more moderately to about 80 °C at 8 weight percent of PE waste, it still surpasses 110 °C at that point. This implies that the asphalt binder’s thermal stability—or its capacity to tolerate greater temperatures before softening—is more significantly influenced by PE waste.

When a needle is introduced into a mixture, the penetration test empirically assesses the consistency of the material. The penetration test was conducted on both base asphalt and modified asphalt samples containing PET and PE waste (Figure 4b). As PET and PE waste content increases, penetration decreases, suggesting that the binder gets stronger and more resistant to deformation. PET exhibits a slightly greater initial penetration than PE waste; nevertheless, the two curves converge at higher percentages, especially at wt%. The data indicate that the asphalt becomes harder with the addition of both additives, while the penetration values of PE waste decrease more sharply.

The characteristics of an asphalt mixture that contains waste plastic were investigated by M. E. Abdullah et al. [38]. They discovered that the softening point of bitumen is 53 °C when plastic waste comprises 1.5% of its weight, and it may reach 56 °C when plastic waste makes up 6% of its weight. It may be stated that sensitivity to high temperatures will decrease as the softening point increases. This finding demonstrates that the binder’s resilience to heat is enhanced, and its propensity to soften in hot conditions is decreased.

When a typical briquette specimen of asphalt material is subjected to tensile testing (Figure 4c) at a set speed of 50 mm/min and a specified temperature of 10 °C, the ductility of the material is determined by the distance (in cm) to which it stretches before breaking. The two clips are pulled apart at a constant pace without vibration until the briquettes burst. As the proportion of discarded PET increases, the mixture’s ductility falls (Figure 4c). For asphalt modified with PE waste, ductility first rises but then starts decreasing after peaking at about 4 wt%. The ductility of asphalt modified with PET continuously decreases as the percentage rises. Up to 4 wt%, PE waste significantly increases ductility; beyond that, the material becomes more brittle. Conversely, when PET content rises, the asphalt binder’s elasticity steadily decreases. To summarize, the softening point of PE waste is considerably higher than that of PET, and the former exhibits superior initial ductility improvements. However, both materials lessen the asphalt binder’s penetration. These results indicate that, at greater modification percentages, PE waste has a stronger positive impact on mechanical and thermal performance than PET.

H. Yu et al. [39] studied the effect of recycled PE-modified asphalt. They found that as the amount of LDPE and RPE increases, the softening point also tends to rise. When compared to LDPE, RPE exhibits a more notable improvement in its softening point, reaching over 70 °C at 6% content. This is 40% more than that of the base asphalt. The rise in modifier concentration is correlated with a discernible decrease in the ductility of the asphalt. However, when the proportion of LDPE and RPE is less than 6%, the ductility of the modified asphalt is improved.

The impact of recycled polystyrene on asphalt properties was examined by Mousa Bani Baker et al. [40]. Their research revealed that the mean penetration values (mean of three tests for each mixture) decreased as the amount of polystyrene increased. A possible reason for this is increased adhesive forces between the polystyrene and bitumen.

The viscosity of the base asphalt was measured at 135 °C and 425 Pa⋅s; Figure 4 illustrates that the modified asphalt has a greater viscosity than the base asphalt. The viscosity of the modified asphalt is unchanged at 135 °C and 2 wt% polymer content, meaning it has no discernible impact on the base asphalt. Conversely, as the amount of modifier component rises, the viscosity of asphalt treated with PET and PE waste progressively increases. It has been shown that asphalt, in its purest form, has more viscosity when PET is added. PE waste is added up to 4500 Pa⋅s, which causes a considerable rise in viscosity. Consequently, bitumen treated with PE waste has a higher workability than asphalt without PET. When comparing asphalt changed with a compound modifier to asphalt treated with a single modifier, the viscosity of the latter rises considerably. The best asphalt with compound modification is compound-modified asphalt because its high-temperature resistance is reflected in its high-temperature viscosity.

The study by Dalhat et al. [41] examined the effect of PE addition on the essential properties of binders, which is consistent with our findings. Many investigations have found that binders treated with PE have greater softening points and viscosity values, as well as poorer penetration and ductility. For every 2% increase in PE content, the peak temperature of performance grade (PG) increased by one degree. The PE-modified binders were found to have lower non-recoverable creep compliance values and to be more resistant to rutting. Dalhat et al. [41] revealed that the PE-modified asphalt binders were not acceptable for elastic recovery criteria. Therefore, they suggested using elastomer to improve the blend’s elastic properties.

Salman Asrar Ahmad and Malik Shoeb Ahmad [1] investigated the effects of mixing PET with bitumen. To find out how the viscosity of the binders treated with PET changed, a viscosity test was performed at 150 °C. The test showed that bitumen in its purest form has more viscosity when PET is added. PET comprises up to 12% of the total, which causes a considerable rise in viscosity. Consequently, bitumen treated with PET has a higher workability than bitumen without PET.

### 3.3. Results of Rheological Properties of Polymer-Modified Asphalt

**Figure 5:**
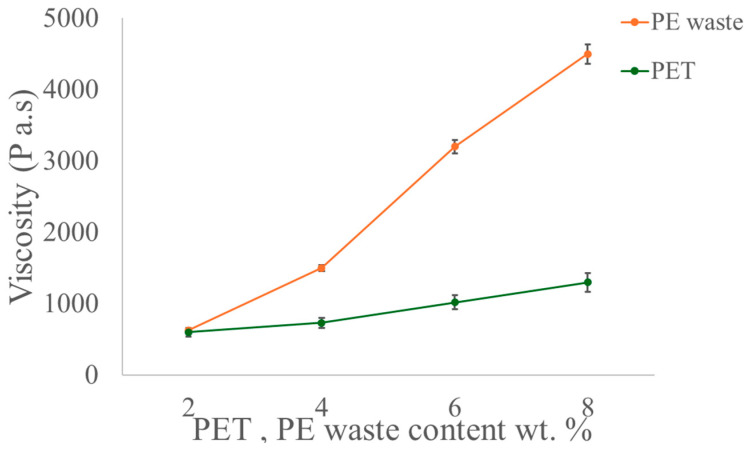
Viscosity of asphalt-modified PET and PE waste.

The DSR test was carried out for the asphalt binder and the polymer-modified asphalt binders at different polymer percentages and test temperatures. The purpose of this text was to illustrate the elastic–plastic behavior of the generated samples. The results are presented in Figure 5. Since polymer additives and asphalt binder interact more effectively, the shear modulus in polymer-modified asphalt tends to rise with increasing polymer concentration. Higher shear modulus values can result from the use of polymers, which can enhance the asphalt mixture’s overall mechanical characteristics, stiffness, and longevity.

Nevertheless, a polymer-modified asphalt’s shear modulus may show a drop with rising temperatures. The viscoelastic properties of asphalt treated with polymers are responsible for this behavior. Shear modulus and stiffness may decrease due to the increased fluidity of polymers and their deformability at higher temperatures. Erda Li et al. [42] reported the outcomes of modifying PE for use in asphalt. As the PE waste dosage is increased, the complex shear modulus progressively rises, reaches a maximum at 5% dosing, and then starts to decrease at 6% dosing. At 46 °C, the complex shear modulus was 116% higher than the basic asphalt. The capacity of asphalt to withstand distortion may be indicated by its complex shear modulus, which suggests that adding PE waste can significantly increase the matrix asphalt’s resistance to deformation at high temperatures.

The same behavior of asphalt-modified polymer was noticed in the study by Elsayed Abdel Bary et al. [43]. The researchers used polyethylene terephthalate waste as a modifier to asphalt and measured the complex shear modulus at different temperatures from 58 °C to 76 °C. The complex shear modulus value for every sample drops as the temperature rises, indicating a reduction in the prepared samples’ rigidity. Thus, increases in temperature cause samples’ elastic behavior to diminish. When the proportion of additional polymer increased, the complex shear modulus value fell at the same temperature. This can be attributed to the fact that each polymer possesses a distinct saturation content with asphalt, beyond which phase separation occurs.

In addition to analyzing the properties of asphalt binder treated with waste plastic polymer, Nuha S. Mashaan et al. [44] showed how adding waste PET might raise the complex shear modulus from 50 °C to 76 °C. Their findings indicate that PET-treated asphalt is stiffer than the unaltered binder, making it more resilient to aging and more durable.

### 3.4. Effect of PET and PE Waste Mixture on the Physical Properties of Asphalt

The practice of modifying asphalt using polymer waste materials, such as polyethylene (PE) and polyethylene terephthalate (PET), is gaining popularity. This approach was found to be effective in enhancing pavement performance and addressing environmental issues. PE waste raises the softening point, improves resilience to extreme heat and heavy traffic, and increases the flexibility of asphalt. Conversely, PET increases rutting resistance and stiffness but, in greater quantities, can decrease ductility, as can be seen in Table 3.

Although the aforementioned polymers have been examined separately, there is a lack of information about their combined effects on asphalt. Combining PET’s rigidity with PE’s flexibility to build more lasting pavements might provide a balanced solution for PE and PET. In an effort to maximize the usage of asphalt under diverse environmental circumstances, this study examines the effects of various combinations of PE and PET on important asphalt parameters, including the softening point, penetration, ductility, viscosity, and shear modulus.

An increase in the percentage of PE waste entails an increase in the softening point. The reason for this is the heightened contribution of PE waste to thermal stability. The blend including 2% PET and 6% PE waste, for instance, has a maximum softening point at 105 °C, making it ideal for high-temperature settings.

Penetration declines with increasing PE waste concentration. This suggests a harder, more deformation-resistant asphalt. With the lowest penetration of 33 dmm, the blend of 6% PE waste and 2% PET is a particularly durable and traffic-resistant material.

Due to its elasticity-enhancing qualities, ductility rises with increasing PE concentration, reaching 80 cm at 6% PE + 2% PET. This combination is the most flexible, making it perfect for cold environments where cracking is a problem. Viscosity increases gradually with increasing PE and PET content, reaching a high of 5500 Pa⋅s for 6% PE + 2% PET. This allows for better workability and resistance to deformation under high temperatures and heavy loads. 

An increase in PE concentration results in the mixture’s stiffness in the complex shear modulus. With the greatest modulus of 8.6 kPa, the blend of 6% PE and 2% PET provides excellent stiffness and deformation resistance, making it appropriate for severe traffic loads. With a remarkable softening point, penetration, and viscosity characteristics, 6% PE + 2% PET seems to be the ideal combination for traffic resistance and stability at high temperatures. In cases where stiffness and flexibility are critical (for instance, in cold areas), 4% PE + 4% PET or 5% PE + 3% PET provides a suitable compromise between ductility and stiffness.

## 4. Mechanical Performance of Asphalt Mixtures

Tensile and compressive tests were conducted to obtain data for mechanical performance, such as compressive strength and tensile strength, for asphalt mixes modified with PE waste, PET, and their combination. The test results summarized in the tables make it possible to compare the efficiency of each method. In order to determine their impact on compressive strength and tensile strength, the mechanical performance of asphalt mixes was evaluated in relation to varying contents of PET and PE waste. By comparing the effects of PET and PE waste separately and in combination, the study aims to ascertain how these polymer modifications influenced the structural integrity and longevity of asphalt mixtures. While the tensile strength provides information about the mixture’s resistance to fracture and distortion under stress, the compressive strength data show how well the material can support heavy loads.

**Figure 6:**
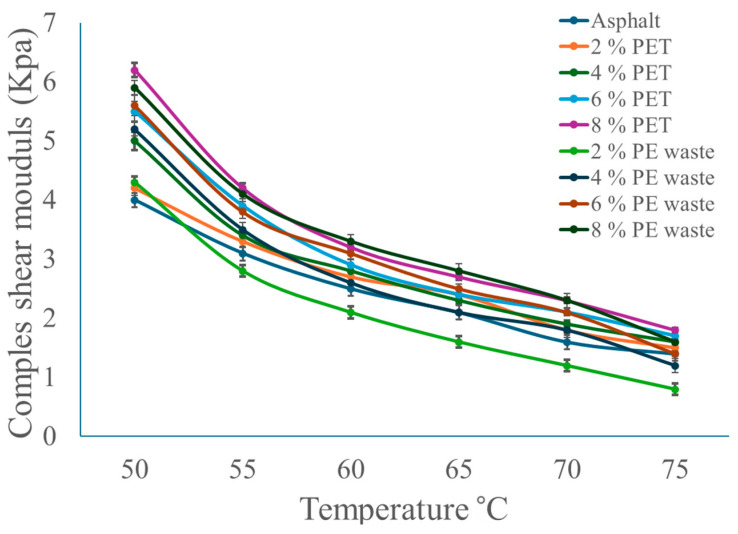
Complex shear modulus results of asphalt-modified polymer.

**Figure 7:**
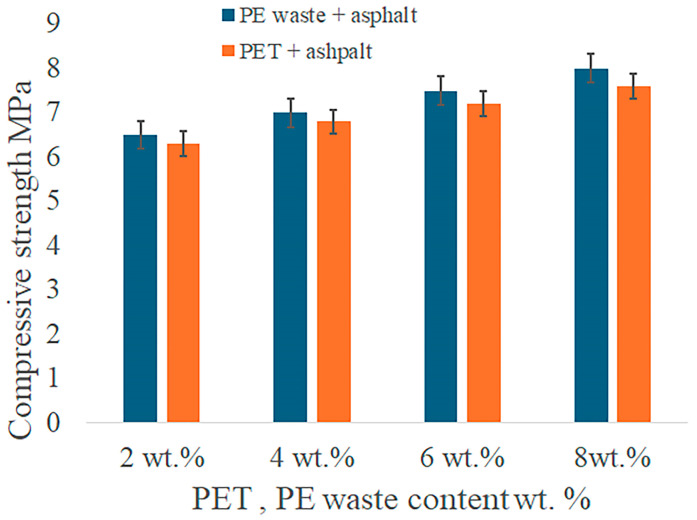
Compressive strength of the PE- and PET-modified asphalt.

The compressive and tensile strength values for asphalt modified with varying percentages of PE waste and PET, as well as mixtures of both, are summarized in the accompanying Figure 6 and Figure 7. The results present a thorough summary of the mechanical gains made possible by polymer modification, emphasizing the best combinations for improved performance in a range of scenarios. It is evident that when the amount of PE waste in the asphalt grows, its compressive strength rises from 5.5 for unaltered asphalt to a maximum of 8.0 MPa at 8% PE waste. PE waste strengthens the asphalt’s elasticity and offers structural support, increasing its resistance to deformation under high loads. Better resistance to tensile pressures and a lower risk of cracking are indicated by the tensile strength, which also rises with PE waste concentration and reaches 1.45 MPa at 8% PE waste. 

**Figure 8:**
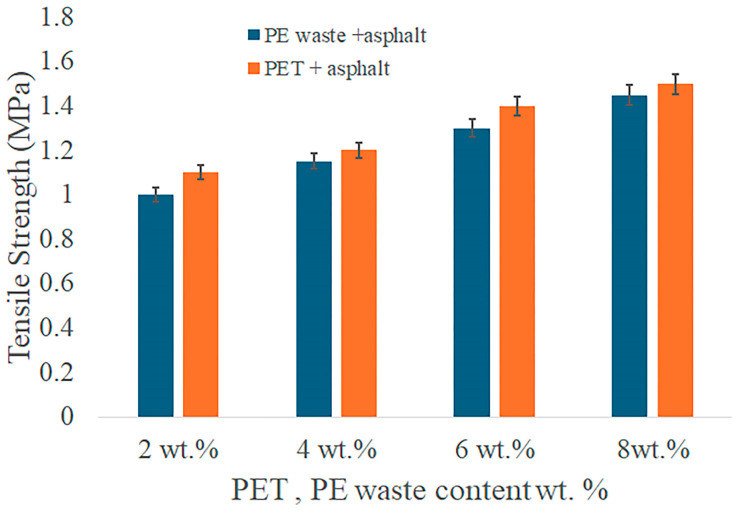
Tensile strength of the PE- and PET-modified asphalt.

With a maximum of 7.6 MPa at 8% PET, PET modification boosts compressive strength to a slightly smaller extent than PE waste. PET adds rigidity, albeit not to the same degree as PE waste, in terms of flexibility. Additionally, PET performs better in tensile strength, reaching 1.50 MPa at 8% PET (Figure 8). These indicators suggest strong resistance to cracking and tensile stresses, making the mixture suitable for high-traffic conditions and cold climates.

**Figure 9:**
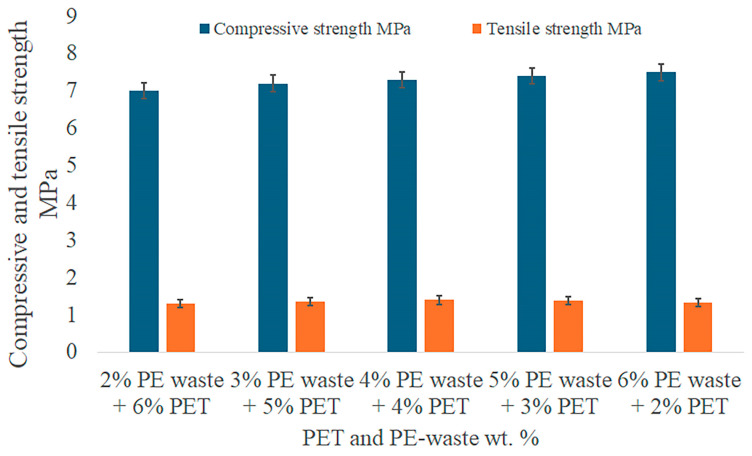
Tensile and compressive strength of asphalt-modified PE waste and PET.

Figure 9 shows the effects of adding PET and PE waste on the mechanical qualities of asphalt. According to the findings, asphalt mixes can achieve the best possible balance between compressive and tensile strength by utilizing the complimentary properties of both polymers. The maximum compressive strength (7.5 MPa) is obtained when 6% PE waste and 2% PET are combined since the stiffness of PET and the flexibility of PE waste improve load-bearing capability. It is advised to blend the two polymers equally for tensile performance, as the blend of 4% PE waste and 4% PET shows the highest resistance to tensile stress (1.40 MPa). This concentration lowers the possibility of cracking and deformation. These results demonstrate how asphalt’s mechanical qualities may be greatly improved by adding PET and PE waste in appropriate proportions. The studied materials enhance the durability and compatibility of asphalt under severe environmental and traffic conditions.

Low-density polyethylene (LDPE) and recycled polyethylene (RPE) were studied by Yu et al. [39] for usage in asphalt modification. The authors found that the compressive strength of RPE-modified asphalt was 8.0 MPa at 6% PE, which was somewhat greater than our finding of 7.5 MPa with 6% PE + 2% PET. Nevertheless, our research showed that the combination of PET and PE results in a more balanced performance across both compressive and tensile strength, indicating higher overall adaptability for various environmental conditions [39].

Vargas et al. [45] conducted a critical investigation of the performance of asphalt treated with waste polymers, such as PE. The analysis confirmed that plastic-treated asphalt was more resilient to rutting and cracking. Nevertheless, the softening threshold did not reach the level observed in our research. Their findings demonstrated that the asphalt modified by both PE and PET had a softening point of 105 °C. In turn, our combination had a softening point of 70 °C, suggesting its better resilience to high temperatures.

The characteristics of PET-modified asphalt were investigated by Choudhary et al. [46]. Their findings showed that the tensile strength of PET-modified asphalt was enhanced to 1.50 MPa when 6% PET was employed, which was marginally greater than the 1.40 MPa tensile strength found in our work using 4% PE + 4% PET. However, the combined modification’s ductility (80 cm for 6% PE + 2% PET) considerably outperforms the ductility seen in asphalt changed alone with PET. Accordingly, the combination of PE and PET used in our study produces a superior balance between stiffness and flexibility [46].

The impact of PET on the asphalt mixture was also examined in the study of M. Pasra et al. [47]. The findings of the tensile test demonstrated that the mixes with PET waste had superior stiffness and elastic region than the mixtures without PET. When PET waste was added to the mixture, the peak tensile stress increased from 45.2% to 96.8%, which was substantially higher compared to the mixture containing no PET waste. The combination with PET waste exhibited greater indicators (56.09% vs. 157.18%) than the mixture without PET waste.

## 5. Statistical Comparison of Experimental Results

To determine whether the differences in asphalt properties (such as compressive strength, tensile strength, softening point, penetration, and ductility) are statistically significant across various combinations of PET and PE waste, this study uses ANOVA (Analysis of Variance). For this study, the significance threshold is set at 0.05 (α = 0.05). This makes it possible to ascertain if variations in asphalt performance metrics (specifically, tensile and compressive strength) between different PET and PE waste concentrations and combinations are merely random fluctuations or represent statistically significant changes. The assumptions of independence, homogeneity of variances, and normality were examined and found to be met prior to ANOVA. 

Before conducting ANOVA, the following hypotheses were examined:

1. Normality: The Shapiro–Wilk test is used to determine the normality of data distribution. In this test, a *p*-value larger than 0.05 indicates that the data exhibit a normal distribution;
2. Independence of Observations: Levene’s test is employed to assess the homogeneity of variances or the equality of variances between the various groups. A *p*-value larger than 0.05 indicates that the variances in different groups are identical;
3. Observational independence is ensured by random sampling, which verifies that observations made within and across groups are unrelated to one another.

In this study, various combinations of PE waste and PET proportions served as the groups (or treatments) for the ANOVA test: 2% PE + 6% PET; 3% PE + 5% PET; 4% PE + 4% PET; 5% PE + 3% PET; 6% PE + 2% PET. Replica data (at least three data points for each combination) for the following parameters were utilized to perform ANOVA: tensile strength (MPa), softening point (°C), penetration (dmm), ductility (cm), and compressive strength (MPa). The ANOVA analysis (Table 4) of each parameter using the sample data revealed that the following *p*-values and F-values are approximate findings. Since 0.05 is the critical value for *p*, any *p*-value below this threshold denotes a significant difference between the groups.

Compressive Strength: The *p*-value of 0.002 suggests a substantial variation in compressive strength across the various PE waste and PET combinations. The F-value of 6.32 indicates that different combinations result in different compressive strengths.

Tensile Strength: Tensile strength also exhibits significant variations between the groups, with a *p*-value of 0.008 for this parameter. The mixture containing 4% PE waste and 4% PET demonstrates the maximum tensile strength, indicating the effect of the polymer combination.

Softening Point: There are very significant variations in the softening point between the combinations, as indicated by the extremely low *p*-value (0.003) and high F-value (35.84). The combination containing 2% PET and 6% PE waste has the highest softening point, making it the most temperature-resistant.

Penetration: There are significant variations in penetration values across the groups, as shown by the *p*-value of 0.003. The toughest mixture is the one with the lowest penetration, namely, the mixture containing 6% PE waste and 2% PET. 

**Figure 10:**
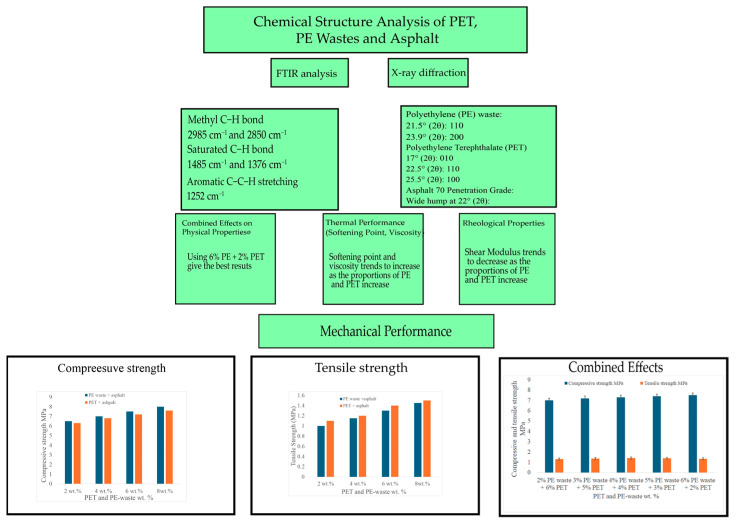
Results of using PE waste and PET as modifiers with asphalt.

Ductility: Significant variations in ductility are also evident based on the F-value (17.45) and *p*-value (0.002). When compared to other combinations, the combination of 6% PE waste and 2% PET offers maximum ductility or higher flexibility. Figure 10 illustrates the results of all tests.

## 6. Conclusions

This study examined the combined impacts of specific modifiers, such as polyethylene terephthalate (PET) and polyethylene (PE) waste materials, on the mechanical, thermal, and rheological characteristics of asphalt. The results showed that mixing these two polymers greatly improves asphalt’s performance, maximizing its softening point, penetration, ductility, and compressive and tensile strength. The combination containing 6% PE waste and 2% PET exhibited the maximum compressive strength of 7.5 MPa, whereas the mixture containing 4% PE waste and 4% PET had the best tensile strength of 1.40 MPa.

The softening point of the asphalt increased in proportion to the concentration of PE waste, with the 6% PE + 2% PET combination having the greatest softening point of 105 °C, which indicates improved resilience to high temperatures. These thermal characteristics were observed in the asphalt. As the PE waste percentage grew, the penetration value dropped; the combination with the highest hardness and resistance to deformation had the lowest penetration value, 33 dmm. The blend of 6% PE waste and 2% PET had the maximum ductility, measuring 80 cm. This indicates superior flexibility, a crucial factor in preventing fractures, particularly in areas characterized by lower temperatures.

The novelty of this work lies in the combined utilization of PET and PE, a practice that has not been extensively explored in previous studies. This research offers fresh insights into the synergistic effects of PET and PE waste, whereas previous studies have primarily focused on examining the individual impacts of each material. The study addresses the problem of managing plastic waste and provides a practical solution to enhance mechanical and thermal performance by optimizing the proportions of these polymers. Thus, the improved ductility, penetration, and softening point of the modified combinations further indicate their suitability for application in harsh environmental conditions.

### Limitations

Although this study offers insightful information, it has certain limitations. First, the testing was carried out in a controlled laboratory setting, which might not accurately represent the climatic and traffic circumstances seen in the real world. Second, the polymer-modified asphalt mixes’ long-term durability was not assessed, particularly with regard to aging, UV resistance, and water susceptibility. Furthermore, this study failed to cover the economic viability of expanding the manufacture and use of such modified asphalt mixes on a broad scale.

Recommendations for Future Studies:

1. Field Testing: To confirm laboratory results and evaluate the long-term performance of PET and PE waste-modified asphalt mixes under actual traffic loads and environmental circumstances, future research should include field testing of these materials;
2. Durability Studies: To guarantee long-term pavement durability, more investigation is required into the aging, UV resistance, moisture susceptibility, and freeze–thaw resistance of polymer-modified asphalt;
3. Economic Feasibility: Further studies should examine the energy needs and environmental effects of producing and implementing PET and PE waste-modified asphalt on a broad scale. A cost–benefit analysis is also required;
4. Other Polymer Combinations: To maximize asphalt performance in various climates and traffic situations, it is necessary to examine the impacts of mixing PET and PE waste with other polymers or waste materials;
5. Variations in Climatic and Traffic Conditions: It is important to study the effect of different weather conditions, including high and low temperatures. The impact of heavy loads on the performance of modified asphalt with PE waste and PET is also an interesting issue to explore;
6. Material and Microstructure: In future studies, it is necessary to explore how polymer fillers are distributed within the asphalt material and study the microstructure of the samples. These data could enable a deeper and more comprehensive understanding of the material’s behavior.

## Figures and Tables

**Table 1:** Physical properties of 70 penetration asphalt.

Material	Properties	Result
Asphalt 70 PEN	Penetration at 25 °C (0.1 mm)	66 ± 2
	Softening point (°C)	49 ± 1
	Ductility at 25 °C (cm)	100 ± 5
	Viscosity at 135 °C	425 ± 10

**Table 2:** Physical properties of PET and PE waste.

Material	Properties				
	Color	Density (g/cm^3^)	Melting Point (°C)	Compressive Strength MPa	Tensile Strength MPa
PET	White	1.35 ± 0.02	255 ± 2.5	75 ± 0.9	45 ± 0.54
PE waste	White	0.57 ± 0.003	135 ± 1.2	79 ± 0.92	49 ± 0.56

**Table 3:** Effects of PE waste and PET combinations on asphalt properties.

Proportion (PE Waste + PET)	Softening Point (°C)	Penetration (dmm)	Ductility (cm)	Viscosity (Pa⋅s)	Complex Shear Modulus (G) (kPa)
2% PE waste + 6% PET	85 ± 1.5	45 ± 0.5	60 ± 0.62	4700 ± 13.5	7.2 ± 0.001
3% PE waste + 5% PET	90 ± 1.8	40 ± 0.5	65 ± 0.62	4900 ± 13.6	7.4 ± 0.001
4% PE waste + 4% PET	95 ± 1.8	38 ± 0.4	70 ± 0.63	5100 ± 14.5	8.1 ± 0.002
5% PE waste + 3% PET	100 ± 1.9	35 ± 0.4	75 ± 0.65	5300 ± 14.6	8.3 ± 0.008
6% PE waste + 2% PET	105 ± 1.9	33 ± 0.31	80 ± 0.68	5500 ± 15.3	8.6 ± 0.008

**Table 4:** ANOVA results.

Parameter	F-Value	*p*-Value	Significance
Compressive Strength	6.32	0.002	Significant Difference
Tensile Strength	4.57	0.008	Significant Difference
Softening Point	35.84	0.003	Significant Difference
Penetration	14.27	0.003	Significant Difference
Ductility	17.45	0.002	Significant Difference

## Data Availability

The original contributions presented in the study are included in the article, further inquiries can be directed to the corresponding authors.
